# Supplementary figures and images for: Weakly supervised veracity classification with LLM-predicted credibility signals
Source: EPJ Data Sci. 2025 Feb 21;14(1):16. doi: 10.1140/epjds/s13688-025-00534-0 (PMC11845407; doi:10.1140/epjds/s13688-025-00534-0)

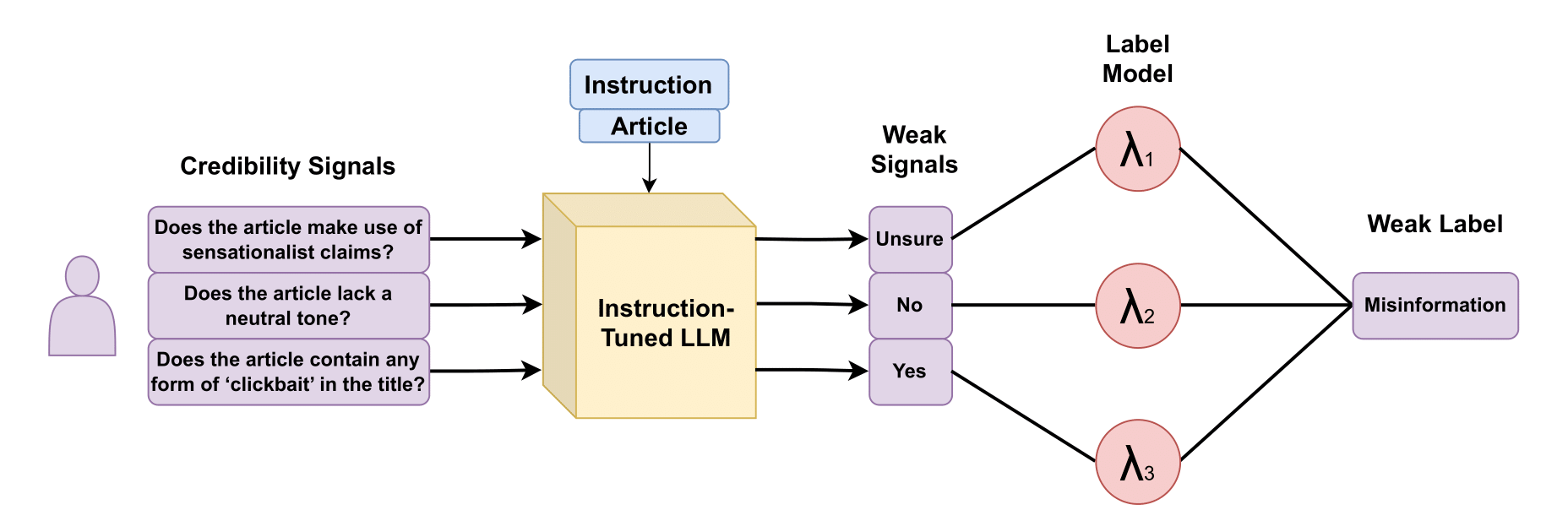

Supplement: Supplementary file 1 — (ZIP 110 kB) [file 13688_2025_534_MOESM1_ESM.zip › PASTEL-main/diagram.png]
